# Using Interleukin 6 and 8 in Blood and Bronchoalveolar Lavage Fluid to Predict Survival in Hematological Malignancy Patients With Suspected Pulmonary Mold Infection

**DOI:** 10.3389/fimmu.2019.01798

**Published:** 2019-08-02

**Authors:** Stephen A. Rawlings, Sven Heldt, Juergen Prattes, Susanne Eigl, Jeffrey D. Jenks, Holger Flick, Jasmin Rabensteiner, Florian Prüller, Albert Wölfler, Peter Neumeister, Heimo Strohmaier, Robert Krause, Martin Hoenigl

**Affiliations:** ^1^Division of Infectious Diseases, Department of Medicine, University of California, San Diego, San Diego, CA, United States; ^2^Division of Pulmonology, Medical University of Graz, Graz, Austria; ^3^Section of Infectious Diseases and Tropical Medicine, Medical University of Graz, Graz, Austria; ^4^Department of Medicine, University of California, San Diego, San Diego, CA, United States; ^5^Clinical Institute of Medical and Chemical Laboratory Diagnostics, Medical University of Graz, Graz, Austria; ^6^Division of Hematology, Medical University of Graz, Graz, Austria; ^7^Center for Medical Research, Medical University of Graz, Graz, Austria; ^8^BioTechMed-Graz, Graz, Austria

**Keywords:** hematologic malignancy, invasive mold infection, interleukin-6, interleukin-8, prognosis

## Abstract

**Background:** Molds and other pathogens induce elevated levels of several cytokines, including interleukin (IL)-6 and IL-8. The objective of this study was to investigate the prognostic value of IL-6 and IL-8 as well as fungal biomarkers in blood and bronchoalveolar lavage fluid (BAL) for overall survival in patients with underlying hematological malignancies and suspected mold infection.

**Methods:** This cohort study included 106 prospectively enrolled adult cases undergoing bronchoscopy. Blood samples were collected within 24 h of BAL sampling and, in a subset of 62 patients, serial blood samples were collected up until 4 days after bronchoscopy. IL-6, IL-8, and other cytokines as well as galactomannan (GM) and β-D-glucan (BDG) were assayed in blood and BAL fluid and associations with overall mortality were assessed at the end of the study using receiver operating characteristic (ROC) curve analysis.

**Results:** Both blood IL-8 (AUC 0.731) and blood IL-6 (AUC 0.699) as well as BAL IL-6 (AUC 0.763) and BAL IL-8 (AUC 0.700) levels at the time of bronchoscopy were predictors of 30-day all-cause mortality. Increasing blood IL-6 levels between bronchoscopy and day four after bronchoscopy were significantly associated with higher 90-day mortality, with similar findings for increasing IL-8 levels. In ROC analysis the difference of blood IL-8 levels between 4 days after bronchoscopy and the day of bronchoscopy had an AUC of 0.829 (95%CI 0.71–0.95; *p* < 0.001) for predicting 90-day mortality.

**Conclusions:** Elevated levels of IL-6 and IL-8 in blood or BAL fluid at the time of bronchoscopy, and rising levels in blood 4 days following bronchoscopy were predictive of mortality in these patients with underlying hematological malignancy who underwent bronchoscopy for suspected mold infection.

## Introduction

Patients with hematologic malignancies are immunocompromised with increased rates of hospital admissions and, in particular, admissions to the intensive care unit (ICU) (1). Factors that increase the risk of admission to the ICU include pneumonia caused by invasive mold infections (IMI) or other pathogens (2). IMI, including invasive aspergillosis (IA), are associated with high morbidity and mortality among patients with underlying hematological malignancies (3–7). Prognosticating survival in patients at risk for IMI remains difficult (8), although prompt identification of those most at risk for severe complications and death and early initiation of antifungal or other anti-infective therapy could lead to better outcomes (9).

*Aspergillus* spp. have been shown to induce T-helper cell subsets resulting in elevated levels of several cytokines (10, 11) and recent studies have indicated—after adjusting for multiple covariates also associated with higher cytokine levels—that particularly Interleukin (IL)-8 and IL-6 may show promise as diagnostic markers (12, 13). Our own work suggests that elevated levels of IL-8 in patients presenting with suspected pulmonary infection have excellent specificity (>90%) for detecting IMI (14), however whether these cytokines may also predict overall mortality and whether serial measurement of this cytokines may increase their prognostic potential remains unknown.

The objective of this analysis was to determine the potential of variations in IL-6 and IL-8, as well as established fungal biomarkers, to predict overall mortality in patients with underlying hematological malignancies and suspicion of pulmonary mold infection in a setting that uses mold-active prophylaxis.

## Materials and Methods

This prospective cohort study comprised paired routine serum and BAL samples obtained on the same day from cases with underlying hematological malignancies who underwent routine bronchoscopy due to suspicion of pulmonary mold infections. The decision was based on suspicious or non-specific radiological findings in chest computed tomography, with or without clinical laboratory findings including fungal biomarker levels. Investigators had no influence on clinical interventions (e.g., bronchoscopy) and treatment of the enrolled patients. The diagnostic potential of several biomarkers and cytokines measured in same day BAL and blood samples obtained as part of this cohort study for diagnosing IMI has been previously published (13, 14). However, the present analysis focuses on the overall prognostic potential of several cytokines and biomarkers in clinical outcomes of IMI. Approximately halfway through the study, the protocol was modified to include permission to use routinely-collected surplus plasma samples stored in the hospital laboratory for 4 days following collection. These longitudinal samples have not previously been published.

IA and IMI were graded in accordance with the revised criteria by the European Organization for Research and Treatment of Cancer Invasive Fungal Infections Cooperative Group (EORTC) and the Mycoses Study Group of the National Institute of Allergy and Infectious Disease (MSG) (15).

### Study Cohort

Participants undergoing bronchoscopy were prospectively enrolled at the Medical University of Graz, Graz, Austria, between April 2014 and July 2017. Key inclusion criteria were (i) adult patients with (ii) underlying hematological malignancy who were (iii) at risk for IMI according to the attending clinicians discretion (e.g., febrile neutropenia, induction chemotherapy for acute myeloid leukemia, allogeneic stem cell transplantation), had (iv) a BAL sample obtained due to clinical suspicion of pulmonary infection, and (v) an order of fungal biomarkers from BAL [i.e., galactomannan (GM) and in a subset also panfungal or Mucorales specific polymerase chain reaction]. All patients who met inclusion criteria between April 2014 and July 2017 and signed an informed consent were included in the cohort. After informed consent was obtained, serum and whole blood samples were collected within 24 h of bronchoscopy. In the last 62 patients enrolled in the study, serial daily plasma samples were also obtained from blood samples drawn as part of routine clinical care from 4 days before bronchoscopy until 4 days after bronchoscopy. Because Investigators had no influence on blood sample drawn besides the same-day samples, sample size varied between 2 and 9 plasma samples per case. These were stored at 4°C for up to 4 days before processing for storage at −70°C and analysis.

### Biomarker Testing

Conventional culture as well as BAL and serum GM concentrations (Platelia enzyme immunoassay; Bio-Rad Laboratories, Vienna, Austria) were prospectively determined in clinical routine at the Medical University of Graz. Given that the vast majority of patients received mold-active antifungals at the time of bronchoscopy, cut-offs of 0.5 GM optical density index (ODI) where used for serum and BAL, following previous evidence that the 0.5 ODI cutoff is preferable in patients on mold-active antifungals (16).

β-D-glucan (BDG) testing was performed in part prospectively and in part retrospectively at the Medical University of Graz, using the commercially available Fungitell^®^ assay (Cape Cod Diagnostics, Falmouth, MA, USA) with an adopted protocol suitable for use on a routine BCS XP^®^ coagulation analyzer, as described previously (17). BDG testing was only performed in serum samples, as BAL BDG testing yields very low specificity due to non-pathogenic *Candida* colonization in the lungs and high BDG values (18–20). For serum BDG we used the recommended cut-off of ≥ 80 pg/mL to define positivity.

All blood (i.e., serum and plasma) and BAL isolates used in this study were frozen to −70°C after processing and stored for batched analysis. IL-6 and IL-8 concentrations were determined in serum, plasma, and BAL samples at the Center for Medical Research of the Medical University of Graz, Austria, between 09/2016 and 10/2017 with a personalized ProcartaPlex^®^ immunoassay (eBioscience, Vienna, Austria) as previously described (13).

Investigators measuring biomarkers and cytokine levels were blinded toward clinical and demographic information of the patients.

### Assessing Mortality

All patients enrolled in the study were followed clinically on initial admission and their medical records were reviewed after discharge. Autopsies were not routinely performed or requested as part of the study and therefore the absolute rate of autopsies on patients in the study was very low—likely reflecting the low cultural predilection for autopsy in the study country.

Our study was conducted in accordance with the Declaration of Helsinki, 2013, Good Clinical Practice. The study protocol was approved by the local ethics committee, Medical University Graz, Austria (EC-numbers 25-221 and 23-343), and registered at ClinicalTrials.gov (Identifier: NCT02058316 and NCT01576653). Informed consent was obtained from all study participants. Statistical analysis was performed using SPSS, version 25 (SPSS Inc., Chicago, IL, USA). For continuous data, including cytokine levels, receiver operating characteristic (ROC) curves analyses were performed and area under the curve (AUC) values are presented including 95% confidence intervals (95% CI) for the 30-, 90-, and 180-day overall mortality outcomes (*p*-values were not corrected for multiple comparisons). Optimal cut-offs for cytokines discriminating in patients who died within 30 days vs. those who survived were calculated using the Youden's index. Two-sided *p*-value < 0.05 was taken as cut-off for statistical significance.

## Results

### Study Cohort

In total, 122 participants undergoing bronchoscopy were prospectively enrolled between April 2014 and July 2017. A total of 16 cases had to be excluded due to the following reasons: (i) same day blood samples (i.e., collected within 24-h) were not available (*n* = 13); (ii) BAL volume after routine testing was insufficient for further diagnostic work up within the study protocol (*n* = 2); (iii) hematological malignancy was suspected but not confirmed because of mortality within days of admission (*n* = 1). After exclusion of these 16 cases, 106 patients remained in the final analysis. Patients' characteristics are displayed in Table 1. Mortality was 16% (17/106) at 30 days, 27.4% (29/106) at 90 days, and 42.5% (45/106) at 180 days after study enrollment and bronchoscopy. In those with mold infections, 30-day mortality was 36% for probable/proven IA, 16% for possible IA and 13% for those without evidence for IA (for all probable/possible/no IMI 33, 12, and 13%, respectively).

**Table 1:** Demographic data, underlying diseases, and infections in cases who died within 30 and 90 days after bronchoscopy vs. those who survived.

**Demographic data, underlying diseases and other characteristics at the time of sampling (*****n*** **= 106)**		**Mortality at day 30 (*n* = 17)**	**Survival at day 30 (*n* = 89)**	*p*-value^*^	**Mortality at day 90 (*n* = 29)**	**Survival at day 90 (*n* = 77)**	*p*-value^*^
Sex	Female	6 (35%)	47 (53%)	>0.2	10 (34%)	43 (56%)	0.050
	Male	11 (65%)	42 (47%)		19 (66%)	34 (44%)	
Age [years]	Median (range)	55 (33–66)	59 (26–85)	0.13	56 (27–78)	58 (26–85)	>0.2
Underlying diseases	AML/MDS	8 (47%)	43 (48%)	>0.2	18 (62%)	33 (43%)	>0.2
	NHL	2 (12%)	22 (25%)		4 (14%)	20 (26%)	
	MM	2 (12%)	7 (8%)		2 (7%)	7 (9%)	
	ALL	2 (12%)	9 (10%)		2 (7%)	9 (12%)	
	Others^#^	3 (18%)	8 (9%)		3 (10%)	8 (10%)	
Other conditions	Allogeneic stem cell transplantation	5 (29%)	23 (26%)	>0.2	8 (28%)	20 (26%)	>0.2
	Autologous stem cell transplantation	1 (6%)	9 (10%)	>0.2	1 (3%)	9 (12%)	>0.2
	Graft vs. host disease	3 (18%)	13 (15%)	>0.2	5 (17%)	11 (14%)	>0.2
	Neutropenia (<500 μL) on day of BAL	12 (71%)	34 (38%)	**0.017**	18 (62%)	28 (36%)	**0.017**
	T-Cell Suppressants within 3 months of BAL	5 (29%)	9 (10%)	**0.047**	7 (24%)	7 (9%)	0.055
	Systemic corticosteroid treatment within 14 days of sampling	5 (29%)	25 (28%)	>0.2	8 (28%)	22 (29%)	>0.2
Invasive fungal infections	Probable/proven IMI	6 (35%)	12 (13%)	**0.039**	6 (21%)	12 (16%)	>0.2
	Possible IMI	3 (18%)	22 (25%)	>0.2	9 (31%)	16 (21%)	>0.2
	Probable/proven invasive aspergillosis	4 (24%)	7 (8%)	0.07	4 (14%)	7 (9%)	>0.2
	Antimould prophylaxis^$^/treatment	17 (100%)	69 (78%)	**0.030**	29 (100%)	57 (74%)	**0.002**
Other infections	Positive diagnostic test for relevant bacterial pathogens, pneumocystis or toxoplasma in BAL	3 (18%)	18 (20%)	>0.2	5 (17%)	16 (21%)	>0.2
	Positive diagnostic test for bacterial infections in other samples (blood culture/biopsies/urine) within 14 days of sampling	5 (29%)	19 (21%)	>0.2	9 (31%)	15 (19%)	>0.2
	Positive diagnostic test for viral infections within 14 days of sampling	6 (35%)	32 (36%)	>0.2	10 (34%)	28 (36%)	>0.2

* *Bold indicates p-values that met statistical significance*.

# *Included 5 chronic lymphoid leukemia (CLL); 3 primary myelofibrosis; 2 Hodgkin's lymphoma, and 1 anaplastic anemia*.

$ *Breakdown of antifungal prophylaxis: 19 received voriconazole, 18 received posaconazole prophyaxis at the time of sampling*.

*ALL, acute lymphocytic leukemia; AML, acute myelogenous leukemia; BAL, bronchoalveolar lavage fluid; GvHD, Graft vs. host disease; IA, invasive aspergillosis; IMI, invasive mold infection; MDS, myelodysplastic syndrome; MM, multiple myeloma; NHL, non-hodgkin lymphoma; SCT, stem cell transplantation*.

Overall patients who died within 30 days were more frequently neutropenic at the time of bronchoscopy, had more frequently received T-cell suppressants, had more frequently probable or proven IMI and had received more frequently mold active prophylaxis or treatment at the time of bronchoscopy (Table 1). Of the 17 cases who died within 30 days of bronchoscopy (median 14 days after bronchoscopy, range 1–30 days), autopsy was performed in four cases, revealing progression of acute myeloid leukemia as cause of death in two cases, while cause of death was infectious (organized viral pneumonia, multi organ failure) in the other two cases. Of the other 13 cases who did not undergo autopsy, five had probable IMI, two possible IMI, two viral pneumonias, and each one bacterial pneumonia or systemic bacterial infection. Neutropenia and receipt of mold-active antifungals were also more frequent in those who died within 90 days, in fact every single patient who died within 90 days had received a mold-active antifungal at the time of bronchoscopy while this was 74% of patients who survived to day 90 (*p* = 0.002).

### Prognostic Potential of Blood and BAL Cytokines at the Time of Bronchoscopy

**Figure 1:**
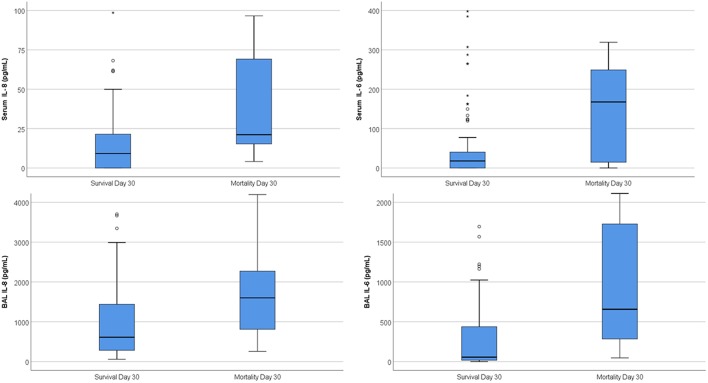
Boxplots of same-day serum and BAL cytokines in those who survived day 30 and those who died. On the boxplot shown here, outliers are identified by different markers. “Out” values (small circle) and “far out” or as SPSS calls them “Extreme values” (marked with a star). SPSS uses a step of 1.5×IQR (Interquartile range).

In ROC curve analysis, serum IL-8 was a significant predictor of 30-day overall mortality, followed by serum IL-6, while serum GM and serum BDG were not significant predictors (Table 2). In BAL IL-6 and IL-8 were significant predictors of overall 30-day mortality, while GM was not (Table 2). Both serum IL-8 and serum IL-6 as well as BAL IL-6 were also significant predictors of 90- and 180-day cumulative overall mortality, although AUCs were lower when compared to 30-day mortality, and significance driven mostly by the predictive potential for 30-day mortality (Table 2). When focusing only on participants who died within 30- and 90-days, or between 90- and 180-days, AUCs were highest for serum IL-8 (AUC 0.578 and 0.592, respectively), followed by serum IL-6 (AUC 0.564 for both; all *p* > 0.2). Boxplots of serum and BAL IL-8 and IL-6 levels in those who died and those who survived are displayed in Figure 1. For prediction of 30-day mortality, optimal cut-offs were serum IL-8 > 13.93 pg/mL (82.4% sensitivity, 61.8% specificity), serum IL-6 > 165 pg/mL (52.9% sensitivity, 87.6% specificity), BAL IL-8 > 1,111 pg/mL (64.7% sensitivity, 69.7% specificity), and BAL IL-6 > 43.95 pg/mL (100% sensitivity, 46.1% specificity).

**Table 2:** Performance of cytokine levels^*^, Galactomannan (GM) and Beta-D-glucan (BDG) in serum and bronchoalveolar fluid (BAL) for differentiating cases who died within 30, 90, and 180 days of bronchoscopy vs. those who survived.

		**Test performance for predicting overall 30 days mortality (17/106)**				**Test performance for predicting overall 90 days mortality (29/106)**				**Test performance for predicting overall 180 days mortality (45/106)**			
**Sample**	**Biomarker or cytokine**	**AUC**	**95% CI**		***p-*value**	**AUC**	**95% CI**		**p-value**	**AUC**	**95% CI**		***p*-value**
			**Lower bound**	**Upper bound**			**Lower bound**	**Upper bound**			**Lower bound**	**Upper bound**	
BAL	BAL GM	0.484	0.318	0.649	0.833	0.477	0.355	0.600	0.720	0.509	0.397	0.620	0.882
	IL-6	***0.763***	***0.663***	***0.863***	***0.001***	***0.687***	***0.570***	***0.805***	***0.003***	***0.620***	***0.509***	***0.731***	***0.036***
	IL-8	***0.700***	***0.574***	***0.826***	***0.009***	0.603	0.483	0.724	0.102	0.536	0.424	0.647	0.534
Serum	Serum GM	0.646	0.477	0.814	0.058	0.571	0.443	0.700	0.259	0.538	0.424	0.653	0.503
	Serum BDG	0.563	0.403	0.724	0.411	0.406	0.279	0.533	0.139	0.446	0.333	0.559	0.346
	IL-6	***0.699***	***0.553***	***0.845***	***0.010***	***0.647***	***0.528***	***0.766***	***0.020***	***0.627***	***0.518***	***0.735***	***0.027***
	IL-8	***0.731***	***0.621***	***0.840***	***0.003***	***0.658***	***0.548***	***0.769***	***0.013***	***0.646***	***0.541***	***0.751***	***0.011***
	IL-10	0.557	0.408	0.706	0.459	0.502	0.380	0.625	0.971	0.514	0.402	0.626	0.808

* *Only cytokines that have shown significant associations with mortality in the previously conducted nested case-control analysis matched for multiple covariates, including neutrophil status, immunosuppressant and concomitant viral and bacterial infections (13), were included in the primary analyses of this study*.

*Significant differences (p <0.05) are bold and italicized (p-values not corrected for multiple comparisons)*.

*AUC, area under the curve; BAL, bronchoalveolar lavage fluid; CI, confidence interval; GM, galactomannan; BDG, beta-D-glucan; IA, invasive aspergillosis; IMI, invasive mold infection; IFN, interferon; IL, interleukin*.

Sub-analyses for 30-day mortality found that (i) among those with possible, probable or proven IMI serum IL-8 (AUC 0.650), BAL IL8 (AUC 0.693), and BAL IL6 (AUC 0.631) had prognostic potential, while serum-IL-6 did not; (ii) among those with bacterial infections, serum IL-8 (AUC 0.685), BAL IL-8 (AUC 0.756), and BAL IL-6 (AUC 0.685) had prognostic potential, while serum IL-6 did not; (iii) among those with viral infections, only BAL levels of IL-8 (AUC 0.734) and IL-6 (AUC 0.677) had prognostic potential, while serum levels did not; (iv) among those with neutropenia at the time of bronchoscopy, serum levels of IL-6 (AUC 0.691) and IL-8 (AUC 0.642) had some prognostic potential while BAL levels had not; in contrast, BAL IL-8 (AUC 0.920) and BAL IL-6 (AUC 0.862) had very strong prognostic potential among non-neutropenic patients; (v) among those on corticosteroids, BAL IL-6 (AUC 0.831) and BAL IL-8 (AUC 0.728) levels had stronger prognostic potential than serum levels; and (vi) among female patients serum IL-8 (AUC 0.862), serum IL-6 (AUC 0.771), BAL IL-8 (AUC 0.812), and BAL IL-6 (AUC 0.801) had all strong prognostic potential.

### Kinetics of Blood IL-6 and IL-8 Before and After Bronchoscopy

**Figure 2:**
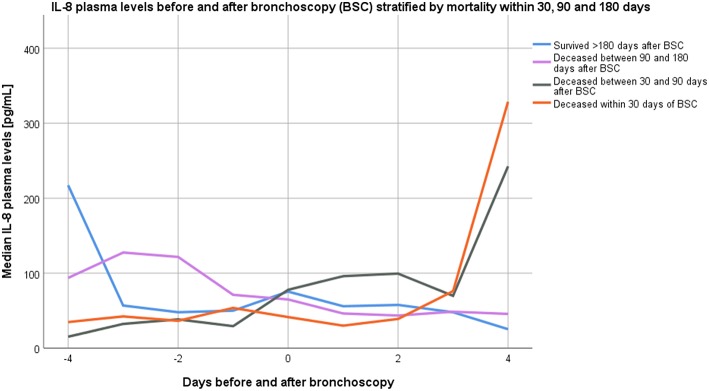
Kinetics of median interleukin-8 plasma levels from 4 days before until 4 days after bronchoscopy stratified by overall mortality categories.

**Figure 3:**
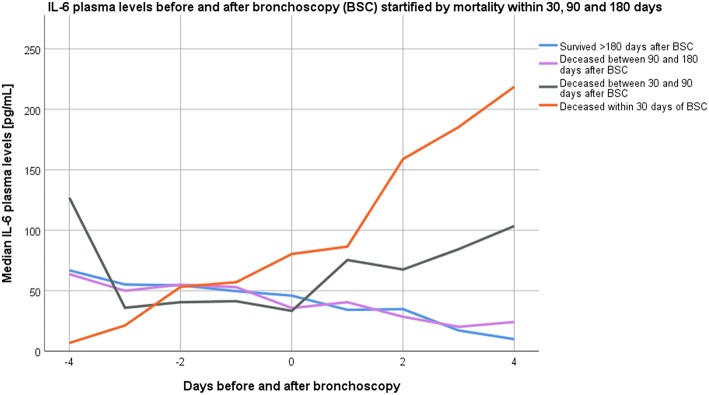
Kinetics of median interleukin-6 plasma levels from 4 days before until 4 days after bronchoscopy stratified by overall mortality categories.

In a subset of 62 participants, serial blood samples were obtained from 4 days preceding to 4 days following bronchoscopy (this particular subset had 30-day mortality of 14%, 90-day mortality of 28%, and 180-day mortality of 50%). Kinetics of serum IL-8 and IL-6 levels stratified by categories of overall mortality are displayed in Figures 2, 3 (23 samples tested for Day −4, 42 samples for Day −3, 46 samples for Day −2; 53 samples for Day −1; 62 samples for Day 0; 48 samples for Day +1; 52 samples for Day +2; 46 samples for Day +3; 49 samples for Day +4).

Between the day of bronchoscopy and 4 days following bronchoscopy (samples available from 49 participants on both time points), blood IL-6 levels increased in 14/49 (29%) participants, while blood IL-8 levels increased in 19/49 (39%) of participants. Increasing IL-6 levels at day four were significantly associated with higher 90-day mortality rates [7/13 (54%) died vs. 7/36 (19%) with stable or decreasing levels; *p* = 0.031], with similar findings for increasing IL-8 levels 4 days following bronchoscopy [9/18 (50%) died vs. 5/31 (16%); *p* = 0.020]. In ROC analysis, the difference of IL-8 levels between 4 days following bronchoscopy and the day of bronchoscopy had an AUC of 0.829 (95%CI 0.71–0.95; *p* < 0.001) for predicting 90-day mortality (AUC for IL-6 0.686; 95%CI 0.499–0.872; *p* = 0.044). AUC for IL-8 difference to predict 30-day mortality was 0.771 (95%CI 0.578–0.963; *p* = 0.023) and for 180-day mortality 0.680 (95%CI 0.529–0.831; *p* = 0.031); while differences in IL-6 levels were not significant predictors for 30- and 180-day mortality.

## Discussion

We evaluated prognostic potential of IL-6, IL-8, and several fungal biomarkers for overall mortality in a high-risk cohort of hematological malignancy patients (>80% of cases had received mold-active antifungals at the time of sampling) undergoing bronchoscopy for suspected pulmonary mold infection. Two major findings are evident. First, elevated levels of IL-6 or IL-8 in blood or BAL fluid at the time of bronchoscopy was associated with increased 30-day overall mortality. Second, increasing blood levels of IL-8 within the 4 days following bronchoscopy were highly predictive of overall 30- and 90-day mortality.

From prior studies, it is understood that cytokines are centrally involved in protective immunity against *Aspergillus* spp. and other molds (13, 21) and may therefore be used as an early biomarker for risk stratification regarding IA associated mortality (22). In the early stages of invasive aspergillosis (IA), conidia are killed by local alveolar macrophage and IL-8, also known as neutrophil chemotactic factor, is produced by these macrophages as well as neighboring epithelial cells as an important chemoattractant for neutrophils (11, 21). The mechanism of IL-8 increase during IA has also been studied *in vitro* where an up-regulation of gene transcription by *Aspergillus fumigatus* proteases was shown to cause increased release of IL-8 (as well as IL-6, which plays an important role in T cell recruitment) by A549 pulmonary epithelial cells and primary epithelial cells (16). Other studies have shown that *in vitro* opsonization of *Aspergillus fumigatus* conidia with H-ficolin, L-ficolin (17), and M-ficolin (which play essential roles in pathogen recognition and complement activation through the lectin pathway) potentiate IL-8 secretion of A549 lung epithelial cells (12, 18, 19). In line, we have previously shown that significantly better diagnostic performances were observed for serum IL-8 and also serum IL-6 when compared to established blood biomarkers (14).

It is worth noting that a number of other conditions/irritants lead to increased levels of IL-6 and IL-8 in both blood and lung environments. Studies have shown increased levels of IL-6 and IL-8 in patients with tobacco smoke exposure and/or chronic obstructive pulmonary disease (20), asthma (23), and influenza infection (24), suggesting these are relatively non-specific cytokines involved in responses to myriad insults that may be visited upon the lungs. These other conditions, including bacterial infections, may have boosted the prognostic potential of these cytokines in our high-risk cohort where IMI was suspected but only confirmed in a subset of cases, as shown in results of our sub-analyses where cytokines were also predictive of overall mortality in those with bacterial and viral infection. As a limitation, prognostic potential of these cytokines may not be limited to patients with confirmed IMI, but may extend to patients with suspected IMI who subsequently are found to have other infections such as bacterial pneumonia. This may, however, also be considered a strength as it would allow for the use of these cytokines more broadly for treatment stratification in hematological malignancy patients with suspected pulmonary infection. While the clinical value of single measurements of cytokines may be more limited (the optimal cut-off for serum IL-8 yielded 82.4% sensitivity but only 61.8% specificity), serial measurements of these cytokines may be more promising. Overall, studies and clinical trials with larger sample sizes are needed to evaluate the prognostic potential of serial measurements of these cytokines for various subgroups of patients.

## Limitations

Overall, fungal infections are rare in patients receiving anti-mold prophylaxis, with a prevalence of 2–3% (25, 26), and therefore multicenter studies are needed to confirm our findings in larger cohorts. To avoid bias introduced by multiple comparisons and confounding factors, we also had to rely on results from smaller, nested matched case-control analysis for identification of cytokines that were evaluated in the primary analyses of this cohort study. Case-control pairs in this nested analysis were matched for multiple covariates that may affect cytokine levels (27).

Additionally, autopsies were performed only in a very small subset of deceased patients and it is therefore likely that the 90- and 180-day mortality causes were not directly related to the initial reason for bronchoscopy (e.g., suspected pulmonary infection). It is still interesting that elevated levels of cytokines had predictive value for mortality so far ahead, suggesting there may be a component of specific immune dysregulation related to these cytokines playing a role in mortality in patients with underlying hematologic malignancies.

## Conclusion

In conclusion, high blood and BAL IL-8 and IL-6 levels at the time of bronchoscopy and, in particular, increasing cytokine levels over time were predictive of mortality in a cohort of patients with underlying hematologic malignancies presenting with concern for pulmonary infection. These findings suggest it could be possible to create a treatment algorithm incorporating measurement of these cytokines at admission and throughout initial treatment for the purpose of identifying patients who warrant more aggressive treatment (e.g., combination treatment) (28, 29) when IMI is suspected in at-risk individuals.

## Data Availability

The datasets generated for this study are available on request to the corresponding author.

## Ethics Statement

The studies involving human participants were reviewed and approved by Medical University Graz, Austria (EC-numbers 25-221 and 23-343). The patients/participants provided their written informed consent to participate in this study.

## Author Contributions

SR, MH, JP, SE, JJ, and SH designed the study and drafted the manuscript. Data were analyzed by MH and SH. Samples were collected by SH, SE, JR, HF, AW, PN, and FP. Samples were analyzed by HS, RK, FP, JR, SH, SE, and JP. The manuscript was critically revised and important intellectual content provided by RK, HS, HF, JR, FP, PN, and AW. The final version for publication was approved by all authors. All authors agreed to be accountable for all aspects of the work in ensuring that questions related to the accuracy or integrity of any part of the work are appropriately investigated and resolved.

### Conflict of Interest Statement

JP received consulting fees from Gilead. AW received speaker honoraria from Merck. RK received research grants from Merck and served on the speakers' bureau of Pfizer, Gilead, Astellas, Basilea, Merck, and Angelini. MH received research grants from Gilead. The remaining authors declare that the research was conducted in the absence of any commercial or financial relationships that could be construed as a potential conflict of interest.
